# Psychological Framing of Illness: Early Family Trauma and Diagnostic Delay in Adult-Onset Metachromatic Leukodystrophy

**DOI:** 10.1155/crps/4267914

**Published:** 2025-11-17

**Authors:** Moritz Metelmann, Wolfgang Köhler, Georg Schomerus, Sven Speerforck

**Affiliations:** ^1^Department of Neurology, Medical Faculty, University of Leipzig, Leipzig, Germany; ^2^Department of Psychiatry and Psychotherapy, Medical Faculty, University of Leipzig, Leipzig, Germany

**Keywords:** arylsulfatase A, case report, diagnostic delay, family system, frontal syndrome, metachromatic leukodystrophy, trauma

## Abstract

Metachromatic leukodystrophy (MLD) is a rare, autosomal recessive disorder of lipid metabolism characterized by deficiency of arylsulfatase A (ARSA), which leads to an accumulation of sulfatides in central and peripheral nerve system and eventually to progressive demyelination. The adult form of MLD may be misinterpreted as a psychiatric disease, since behavioral signs may precede intellectual decline. Here we report the case of a 53-year-old woman initially admitted to a psychiatric ward with symptoms of depression. The behavioral changes were initially attributed to psychosocial stressors within the family, particularly long-term emotional abuse by the patient's former partner. However, detailed anamnesis with the patient's mother revealed progressive behavioral and cognitive decline, urinary and fecal incontinence, that is, features suggestive of an underlying neurological disorder. Notably, laboratory investigations recommended 6 years earlier had not been performed. Neurological examination revealed signs of a frontal syndrome, bilateral pyramidal tract involvement, and mild polyneuropathy. Magnetic resonance imaging (MRI) demonstrated abnormal white matter signal alterations. Further diagnostic investigations showed reduced serum ARSA activity, elevated urinary sulfatides, and a homozygous pathogenic variant in the *ARSA* gene, confirming the diagnosis of adult-onset MLD. The homozygous mutation indicated parental consanguinity, suggesting early trauma embedded within the family. This case underscores the complexity of diagnosing MLD and emphasizes the importance of integrating psychiatric, neurological, and systemic family perspectives in the diagnostic process of rare and slowly progressing illnesses.

## 1. Introduction

Metachromatic leukodystrophy (MLD; Online Mendelian Inheritance in Man [OMIM] no. 250100) represents a rare, autosomal recessive disorder of lipid metabolism characterized by deficiency of the lysosomal enzyme arylsulfatase A (ARSA) due to mutations in the *ARSA* gene [1]. The ARSA deficiency causes reduced degradation and accumulation of sulfatides and lysosulfatides in the central and peripheral nervous system, which finally leads to progressive demyelination and astrogliosis [2, 3].

Incidence of MLD accounts for 0.6–1.8 per 100.000 live births in Europe [4–6]. Three clinical subtypes are known depending on age at onset: late-infantile, that is, patients younger than 3 years of age; juvenile, that is, patients less than 16 years of age; adult, that is, patients 16 years or older [7]. Slower disease progression is seen in juvenile and adult forms [7, 8].

Diagnosis of the adult form may be deterred from misinterpreting as psychiatric disease, since behavioral signs precede or accompany a decline of intellectual capacities [7, 9, 10].

We describe the case of a 53-year-old Caucasian woman with a history of an emotionally abusive marriage, slowly progressive behavioral changes and cognitive decline for over a decade, which emerged as an adult MLD.

## 2. Case Presentation

The 53-year-old Mrs Z and her mother described emotional abuse by her ex-husband as main reason for admission to our ward in 2020. Mrs Z's husband's aggressive behavior over years aggravated until they got divorced in 2012. Since that time Mrs Z felt shivering and discomfort when she thought about her ex-partner. She felt traumatized by him.

Mrs Z's career as educator was characterized by frequent job changes, leading to at least 14 different jobs in the last 5 years of her professional life upto 2014. Mrs Z's husband continuously urged her to apply for new jobs, and he even wrote applications for her by his own. Mrs Z increasingly got under considerable strain, stressed by the work in daycare centers and took sick leaves. She kept up appearances towards her family by, for example, leaving home in the morning pretending to go for work.

During Mrs Z's stay in the psychiatric ward, her mother, who is also her legal guardian, reported about Mrs Z's difficulties in handling stressful tasks, such as staying in contact with other people or speaking in front of them. Mrs Z had even difficulties to getting up in the morning. Mrs Z was also not able to manage her economy, for example, she bought a large amount of digital versatile discs (DVDs), but used just a few of them, and kept the majority in unopened shopping bags. Mrs Z drank a lot of alcohol in 2010 and 2011. She neglected her hygiene and cleanup of her apartment, which appeared to be progressively littered. Mrs Z seemed to love her daughter, but did not develop ideas for joint activities, so that the child mostly watched TV.

Mrs Z developed urinary incontinence in 2018 and fecal incontinence in 2019, which mostly appeared as a strong motivational deficit to reach the bathroom in time.

In the last 6 months before admission to our ward, the patient increasingly forgot her keys, bag, cell phone, or even appointments with her family. From the mother's point of view, the patient had no insight for the current situation.

### 2.1. Family History

There were no known psychiatric or neurologic diseases in the family. However, with growing confidence in the treatment team the patient's mother disclosed that the patient's father and his ancestors were unknown, since the patient was conceived in a rape of the patient's mother and grew up with her grandparents.

### 2.2. Previous Hospital Stays

In May 2014, Mrs Z was admitted to a psychiatry and neurology department. In neurocognitive testing, she showed deficits in verbal long-term memory, impulsive work behavior, and attention performance. Magnetic resonance imaging (MRI) showed severe leukoencephalopathy. Mrs Z was diagnosed by psychiatrists with a moderate depressive episode, organic affective disorder, and mild cognitive impairment, and she was treated with piracetam, citalopram, and amitriptyline.

Neurologists found additionally a mild sensible, axonal polyneuropathy in neurography. In further extensive diagnostic cerebrospinal fluid (CSF) and blood testing, including neurochemical dementia markers, neuronal, rheumatic, and thyroid autoantibodies as well as microbial testing, that is, borrelia, and syphilis and viral testing, that is, herpes simplex virus (HSV), human immunodeficiency virus (HIV), and varicella-zoster virus (VZV) serology as well as long-chain fatty acids were all within the reference values. An ARSA test had been ordered, probably because the neurocognitive deficits combined with the severe leukoencephalopathy led the doctors to think about leukodystrophies. However, the sample was lost and a reanalysis had been recommended but not performed.

### 2.3. Current Findings

Mrs Z presented with a frontal lobe syndrome with hampered drive, social withdrawal, changed day–night rhythm and indistinct orientation in time and location (video in Supporting Information). Among the neuropsychological tests frontal assessment battery [11] detected dysfunction of verbal fluency and inhibitory control (14 points, below-average). Wechsler Memory Scale (WMS-R) [12] with 18 points were below average and Consortium to Establish a Registry for Alzheimer's Disease (CERAD) [13] showed deficits in all domains except normal mini mental status test [14]. The clock drawing test [15] displayed mild visuospatial deficits (2 points), trail making test (TMT) A and B [16] was below average and the Mehrfachwahl-Wortschatz-Intelligenztest (MWT-A) [17], a test of intelligence quotient (IQ) ended with 16 points, comparable to IQ 73–90.

Neurologic examination revealed a pathologic Luria test [18], with pyramidal tract signs to the lower limbs and a mild symmetric sensible polyneuropathy.

**Figure 1:**
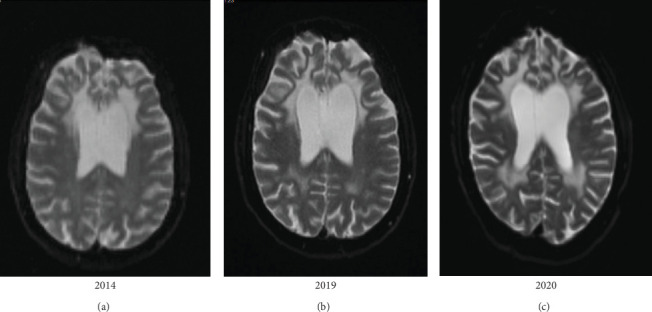
Axial T2-weighted MRI images showing progression of bilateral leukodystrophy white matter involvement in frontal and parieto-occipital lobes from 06/2014 (A) to 08/2019 (B) and 10/2020 (C).

The fluorodeoxyglucose (FDG)–positron emission tomography (PET) revealed a significantly reduced activity distribution in the bifrontal area, including the anterior cingulate cortex, in the high-parietal region and parts of the occipital and temporal cortex. Radiotracer enhancement was distinctly reduced subcortically in basal ganglia and thalamus. MRI displayed a moderate cortical atrophy with strong accentuation on perisylvic and bifrontal areas. In addition, it showed a significant atrophy of bifrontal white matter and extended T2 hyperintense and T1 hypointense white matter lesions with frontal accentuation, which reached the lateral ventricles and the cortex, not including the U-fibers (Figure 1).

CSF analysis indicated a mild disturbance of blood-brain-barrier: white blood cell count 1 cell/mikroL (ref value 0–4), erythrocytes 0 cells/mikroL (ref value ≤ 0), protein 533.0 (+) mg/L (ref value 200–500 mg/L), glucose 4.09 (+) mmol/L (ref value 2.22–3.89 mmol/L), and lactate 2.06 mmol/L (ref value 1.3–2.5 mmol/L). The protein profile did not show any intrathecal synthesis of immunoglobulin G (IgG), IgM, or IgA and the oligoclonal bands were negative. Reiber scheme indicated a mild disturbance of the blood-brain-barrier. In CSF amyloid ß was diminished, while amyloid ß ratio and Tau protein were within normal range (amyloid ß 1–42, 371.7 pg/mL (−); amyloid ß 42/40 ratio, 0.12; phospho Tau protein, <15.6 pmol/mL; Tau protein, 71.5 pg/mL). Resting state-electroencephalography (EEG) showed a slightly slowed basic rhythm and a slight, diffuse dysfunction derived from deeper structures.

In laboratory diagnostics, enhanced level of sulfatides in 24-h urine collection (381.7 nmol/L [normal range 6.0–142.7]) and deficiency of ARSA (0.02 nmol/mg/min [normal range 0.3–1.19]; activity 2.86%) was determined. Genetic analysis revealed a homozygous variant in the *ARSA* gene (chr22:51065802; NM_000487.6: c.257G > A, Arg86Gln), which very rarely occurs: allele frequency in general population 0.00817% according to the Genome Aggregation Database (gnomAD). In conclusion, we diagnosed an adult onset MLD and referred the patient to the leukodystrophy outpatient clinic.

Because of the strong motivational deficit during the stay at our clinics, Mrs Z had been given the antipsychotic drug aripiprazole (5 mg per day). It mildly stabilized the mood of the patient and enhanced the performance — even at the appointment in the outpatient clinic 7 months afterwards.

## 3. Discussion

Progressive cognitive decline and behavioral signs compatible with a frontal lobe syndrome, as shown by our patient, are characteristic clinical signs for the adult form of MLD [19–21]. In the course of disease, neurological symptoms, like spastic paraparesis or ataxia, autonomic failure, like urinary and fecal incontinence as well as mild polyneuropathy are frequently described [22]. Adult MLD patients, such as the case presented here, who predominantly or exclusively show psychiatric symptoms at disease onset, are at risk to be misdiagnosed as having schizophrenia or an affective disorder [23].

In adult MLD cases the residual ARSA activity is about 2%–4% of normal, resulting in a moderate accumulation of sulfatides, which may explain the late onset and slow progress of disease [7]. Like in our patient, MRI in MLD is typically characterized by confluent, symmetric T2-weighted white matter hyperintensity, predominantly in the periventricular regions of the frontal lobes, followed by brain atrophy in late stages of disease [24]. Genetic testing, detecting homozygous variants in *ARSA*-gene, confirms the diagnosis [25, 26].

In our patient, the genetic results shed light on the family system, because homozygosity of the very rare *ARSA* variant suggests consanguinity of the patient's parents. The mother told—with growing confidence during treatment—that the patient had been conceived in a rape. From a systemic family therapy point of view, the long delay of correct diagnosis of this hereditary disease may be partially explained by an anticipated need for protection of the family from the consequences of disclosing a traumatic occurrence within the family.

Recently, ex-vivo gene therapy using a lentiviral vector encoding the human *ARSA* gene in autologous CD34^+^ hematopoietic stem and progenitor cells had been approved for the treatment of early stages of late infantile or early juvenile MLD [27, 28]. Hematopoietic stem cell transplantation can stabilize or delay disease progression in low-progressive juvenile and adult MLD at presymptomatic or very early symptomatic stage [29, 30]. However, there is currently no disease-specific curative treatment for adulthood MLD and management is, therefore, typically palliative [26, 29]. We initiated an increased care level and a nursing home with integrated sheltered workshop after assessment of our occupational therapists and physiotherapists and offered multidisciplinary ambulant care in our leukodystrophy outpatient clinics.

## 4. Conclusion

Our case report may raise awareness for the rare disease of adult MLD, which easily can be misinterpreted as a psychiatric disorder at onset. This case illustrates even more the neuro-psychiatric-psychotherapeutic interrelation, because a superficial psychiatric symptom explanation stabilized the family but led away from a hidden history of violence in the family and, ultimately, from the genetic background of MLD.

## Data Availability

The data that support the findings of this study are available on request from the corresponding author. The data are not publicly available due to privacy or ethical restrictions.

## Supporting Information

Supporting Information Video of patient interview in agreement with the patient and her legal guardian. Additional information: In the verbal fluency test the patient told the following German words: “Fallen, Fuß, Fußball, Flugzeug, Fallschirm, Fallhieb, Fichte” (14:20 min). All words correctly started with “F” and except “Fallhieb” it has been all existing words. The patient persevered the word stem “Fall-“ in the words “Fallen,” “Fallschirm,” and “Fallhieb” as well as the word stem “Fuß-“ in “Fuß” and “Fußball.”
